# Recurrence of Intraocular Inflammation Following an Aflibercept 8-mg Injection in Eyes With a History of Intraocular Inflammation From Faricimab: A Case Report

**DOI:** 10.7759/cureus.86113

**Published:** 2025-06-16

**Authors:** Shiori Sato, Yoichi Sakurada, Yoshiko Fukuda, Yumi Kotoda, Kenji Kashiwagi

**Affiliations:** 1 Department of Ophthalmology, University of Yamanashi, Chuo, JPN

**Keywords:** aflibercept 8mg, age-related macular degeneration, faricimab, intraocular inflammation, macular neovascularization

## Abstract

Vascular endothelial growth factor (VEGF) is critical in various retinal diseases. VEGF inhibitors are a standard treatment for neovascular age-related macular degeneration (AMD). However, intraocular inflammation (IOI) is a major complication after intravitreal administration of second-generation VEGF inhibitors.

We report a case of IOI following treatment with aflibercept 8 mg in an eye with a history of IOI due to faricimab. An 83-year-old man was referred to our clinic and diagnosed with type 1 macular neovascularization (MNV), with a previous treatment history of four doses of ranibizumab, 14 doses of aflibercept (2 mg), and two doses of faricimab. The best-corrected visual acuity (BCVA) in the right eye was 0.3 in the decimal format. Treatment was initiated with an as-needed faricimab regimen after three consecutive monthly injections. Thirteen days after the ninth faricimab injection, the patient complained of blurred vision in the right eye. Mild inflammation, with small keratic precipitates (KPs), was observed in the anterior chamber. After topical dexamethasone administration (four times a day), the anterior chamber inflammation resolved one week after treatment. However, exudation recurred 13 weeks after the ninth faricimab administration; therefore, the intravitreal injection was switched to aflibercept 8 mg. Two days after switching to aflibercept 8 mg, the patient complained of pain and blurred vision in the right eye. As with the ninth faricimab injection, mild inflammation was observed in the anterior chamber of the right eye. One week after subtenon triamcinolone acetonide (STTA) and topical dexamethasone, inflammation disappeared in the anterior chamber. In eyes with a history of IOI, careful attention is needed when switching to aflibercept 8 mg from another second-generation VEGF inhibitor, including faricimab.

## Introduction

Vascular endothelial growth factor (VEGF) plays a critical role in retinal and choroidal diseases, including neovascular age-related macular degeneration (AMD), macular edema secondary to diabetic retinopathy, and retinal vein occlusion [1]. In the early 2000s, ranibizumab, the second commercially available VEGF inhibitor after pegaptanib, revolutionized neovascular AMD treatment [2]. In the ANCHOR and MARINA trials, investigating the efficacy of monthly ranibizumab for neovascular AMD, monthly administration of ranibizumab improved the best-corrected visual acuity (BCVA) in eyes with neovascular AMD, irrespective of the macular neovascularization (MNV) subtype [2]. In the VIEW 1 and 2 studies, aflibercept, administered every eight weeks following three consecutive monthly loading doses, was non-inferior to monthly ranibizumab dosing in improving BCVA over 54 weeks [3].

In 2020, the HAWK/HARRIER trials, comparing the efficacy between brolucizumab and aflibercept, demonstrated that brolucizumab increased BCVA gain non-inferiorly to aflibercept, with more than 50% of eyes maintained at 12-week intervals for 48 weeks [4]. In 2022, faricimab, a bispecific inhibitor of VEGF and Ang-2, became commercially available, and the TENAYA/LUCERNE trials, investigating the efficacy of faricimab compared to aflibercept 2 mg for neovascular AMD, demonstrated that it was comparable to aflibercept 2 mg in terms of visual improvement, with fewer injections [5]. In 2024, the PULSAR study demonstrated that 79%-88% of eyes treated with aflibercept 8 mg maintained a 12- to 16-week treatment interval at a 48-week visit [6]. These drugs have the potential to extend the treatment interval and reduce the physical burden on patients and physicians, and are referred to as “second-generation VEGF inhibitors.” Although the incidence of systemic adverse events is comparable between classic and second-generation VEGF inhibitors, intraocular inflammation (IOI) is a major concern after the administration of second-generation VEGF inhibitors, although a treatment strategy has been established [7-9]. 

We report a case of IOI following aflibercept 8-mg administration in an eye with a history of IOI due to faricimab.

## Case presentation

This case was presented to the University of Yamanashi in July 2023. An 83-year-old man was referred to Yamanashi University Hospital with complaints of visual deterioration in the right eye. The BCVA was 0.3 in the right eye and 0.6 in the left eye. Baseline characteristics are summarized in Table 1.

**Table 1:** Baseline characteristics in both eyes

	Right eye	Left eye
Best-corrected visual acuity (decimal format)	0.3	0.6
Intraocular pressure (mmHg)	12	10
Anterior segment	Normal	Normal
Middle segment	Intraocular lens	Intraocular lens
Posterior pole	Type 1 macular neovascularization	Drusen

**Figure 1:**
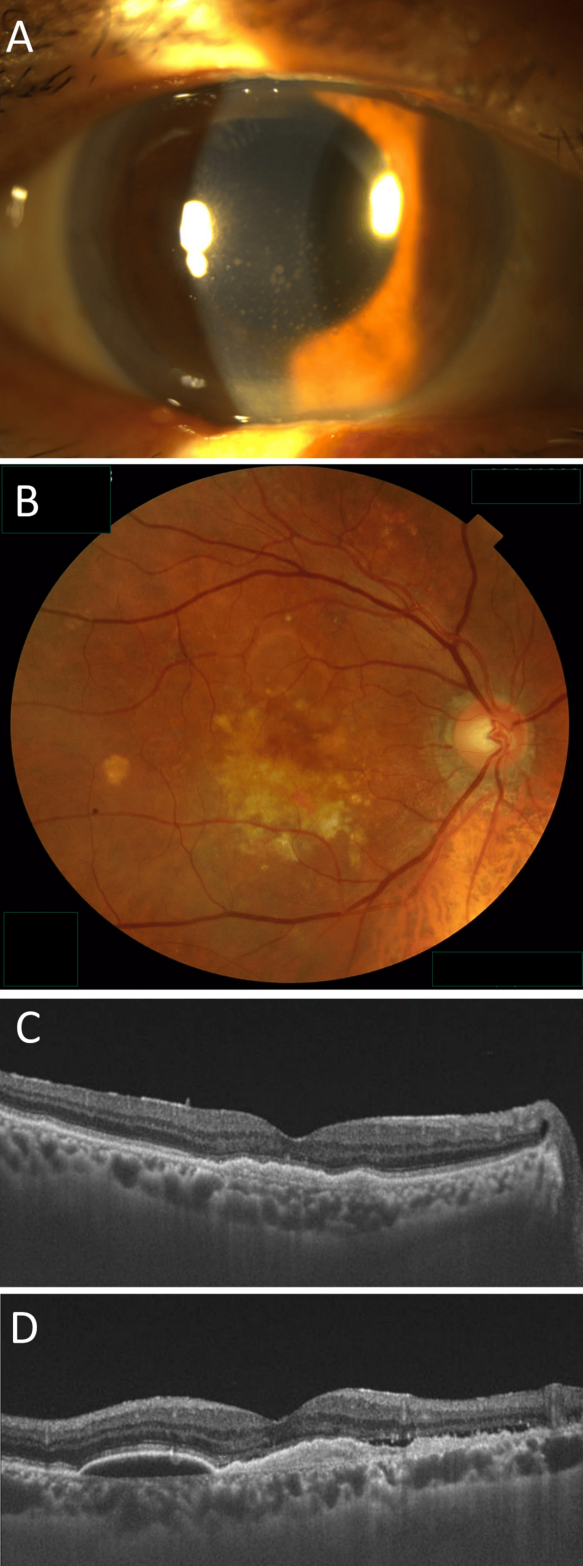
Intraocular inflammation developing 13 days after the ninth faricimab administration (A) In the right eye, keratic precipitates and cells are observed in the inferior cornea and the anterior chamber; (B) color fundus photography reveals macular pigment abnormalities, damage in the macular region, and serous pigment epithelial detachment superior to the macula; (C) a horizontal optical coherence tomography (OCT) scan shows no signs of exudation, with shallow, irregular pigment epithelial detachment (PED); (D) a vertical OCT scan reveals residual subretinal fluid, with shallow, irregular PED and low-height serous PED.

At the initial presentation, a comprehensive ophthalmic examination was performed, including swept-source optical coherence tomography (SS-OCT), fluorescein and indocyanine green angiography, and color fundus photography. The patient was diagnosed with type 1 MNV secondary to AMD in the right eye. He had a history of four intravitreal ranibizumab injections, 14 intravitreal injections of aflibercept 2 mg, and two injections of faricimab. Three monthly intravitreal administrations of faricimab (0.6 mg/0.05 mL) were initially started, and thereafter, additional injections were performed if exudation was seen on SS-OCT. Thirteen days after the ninth faricimab injection, the patient complained of blurred vision in the right eye (Figure 1).

**Figure 2:**
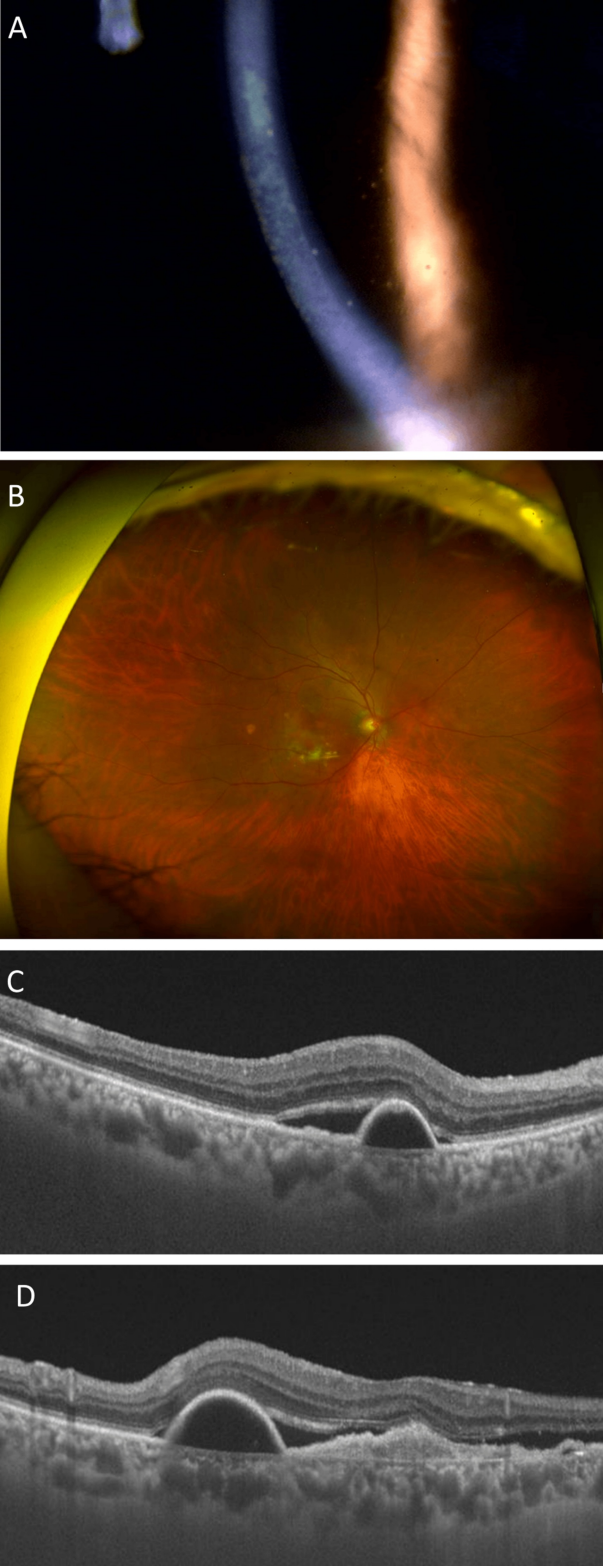
The second intraocular inflammation episode developing two days after aflibercept 8-mg administration (A) In the right eye, keratic precipitates are seen in the magnified inferior cornea; (B) a wide-field color fundus photograph shows no signs of vitritis (vitreous opacity) or retinal vasculitis; (C) horizontal and (D) vertical optical coherence tomography (OCT) scans show serous pigment epithelial detachment with residual subretinal fluid.

Mild inflammation, with small keratic precipitates (KPs), was observed in the anterior chamber of the right eye. No obvious inflammation was observed in the vitreous of the right eye. He was diagnosed with IOI following faricimab administration to the right eye, and topical dexamethasone was initiated four times per day. One week after the treatment, the inflammation in the anterior chamber resolved. Ninety days after the ninth faricimab administration, recurrent exudation was observed on SS-OCT images. The VEGF inhibitor was then switched from faricimab to aflibercept (8 mg). Two days after the administration of 8-mg aflibercept, he complained of blurred vision and pain. BCVA was 0.4 in the right eye (Figure 2).

**Figure 3:**
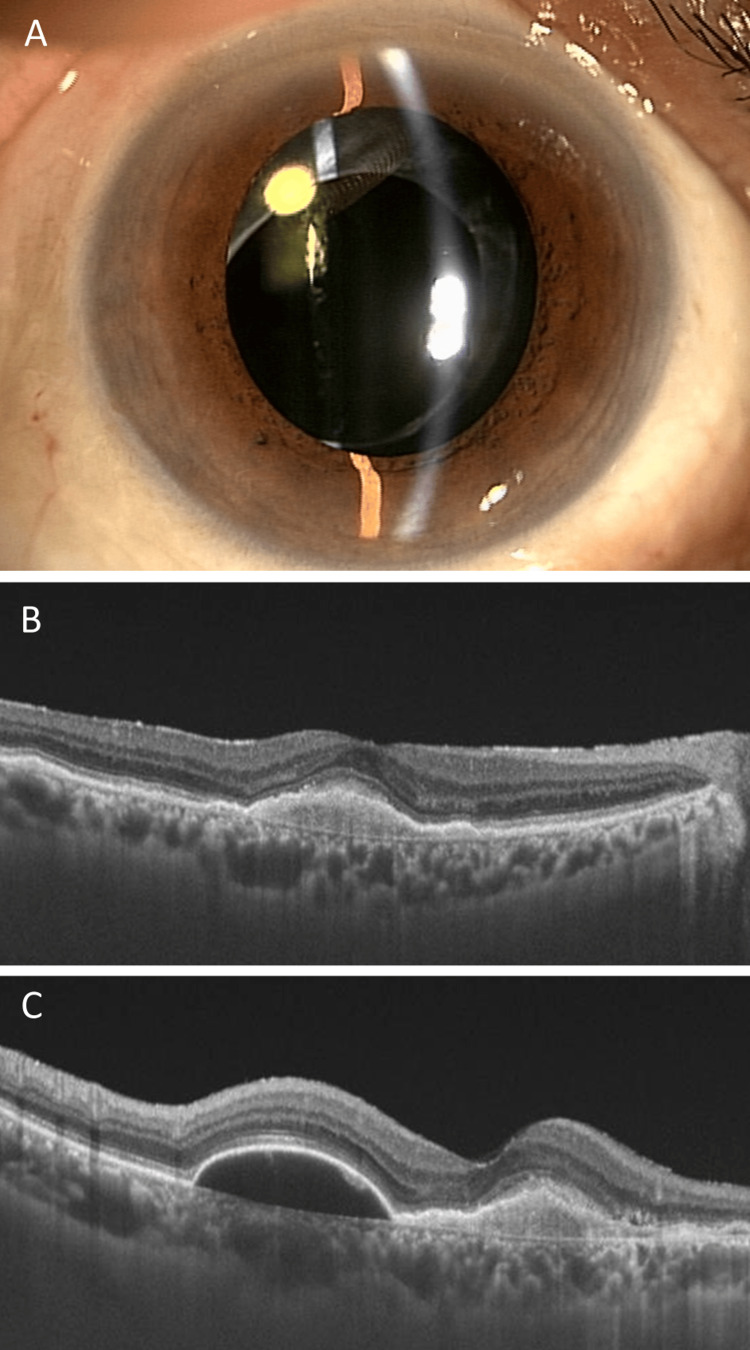
Anterior and posterior ocular imaging one month after the treatment of the second intraocular inflammation (A) In the right eye, keratic precipitates and anterior inflammation resolved; (B) horizontal and (C) vertical optical coherence tomography (OCT) scans show fibrovascular pigment epithelial detachment (PED) and serous PED, respectively, without subretinal fluid.

After the ninth faricimab injection, mild inflammation with small KPs was observed in the anterior chamber of the right eye, and intraocular pressure was 12 mmHg in the right eye; subtenon triamcinolone acetonide (STTA) and topical dexamethasone were administered. One week after the treatment, the inflammation in the anterior chamber disappeared. Figure 3 shows anterior and posterior ocular imaging one month after the treatment of the second IOI.

## Discussion

After the advent of brolucizumab, IOI has become a recognized complication among retinal specialists [10]. The mechanism of IOI has not been fully understood, but the immune complex with a high molecular dose is considered one of the causes of IOI. The incidence of IOI after brolucizumab administration appears to vary in eyes with exudative AMD by ethnicity, with reported rates of 10%-15% in Asian populations and 2%-5% in Caucasian populations [11-13]. Several causes of IOI were proposed. IOIs are exclusively seen when second-generation VEGF inhibitors are administered. This finding suggests that a rapid and excessive reduction of the VEGF level in the vitreous humor might be associated with the onset of IOI. The incidence of faricimab use has not been reported in Asian populations. However, a recent French study demonstrated that the incidence of IOI following faricimab was 0.87% (11/1271); vitritis and small KPs were seen in eight eyes and three eyes out of 11 developing IOI, respectively, within 2-14 days of injection. No cases of retinal vasculitis or vascular occlusions were reported [14]. These findings suggest that IOI related to faricimab may be less frequent, milder in severity, and associated with a more favorable prognosis compared with brolucizumab. In this case, mild inflammation with KPs was observed in the anterior chamber of the right eye, without posterior/intermediate inflammation. The presentation in the anterior chamber was similar to that reported previously. The BCVA (0.3 in decimal format) before the adverse event was maintained after topical dexamethasone treatment.

Aflibercept 8 mg is a novel, second-generation VEGF inhibitor. In a single injection, its molecular dose is higher than that of faricimab [6]. Therefore, it is expected that dry macula sustains for a long interval. Few studies have reported the real-world outcomes of this drug [15]. The PULSAR study reported that IOI due to aflibercept 8 mg was almost 1%, which was comparable to that of 2-mg aflibercept [6]. However, a recent clinical study reported that 8.6% of eyes developed IOI associated with mild retinal vasculitis after 8 mg of aflibercept [16]. Another study reported that eight eyes developed IOI after the administration of 8-mg aflibercept [17]. In this report, most eyes presented with mild vitreous and/or anterior chamber inflammation without retinal vasculitis. Although the clinical presentations differed between the two studies, the inflammation was mild and resolved after local steroid treatment. In this case, inflammation in the anterior chamber after aflibercept 8-mg administration was mild without vitritis and resolved one week after STTA, and BCVA was maintained at 0.4 after switching from faricimab to aflibercept 8 mg. As in previous studies, inflammation was responsive to steroid treatment, and visual deterioration due to IOI was not observed.

When IOI develops once, the same drug should not be administered [18]; therefore, we selected aflibercept 8 mg for recurrent exudation in this case. Indeed, a recent case series reported three cases of mild IOI due to faricimab developing occlusive retinal vasculitis after repeated administration of faricimab [18]. In our case, anterior inflammation recurred after switching to aflibercept 8 mg from faricimab. Both drugs are categorized as second-generation VEGF inhibitors. This implies that careful monitoring should be performed when switching to aflibercept 8 mg from another second-generation VEGF inhibitor in eyes with a history of IOI. Whether the inflammation was due to drug-specific effects or immune sensitization from prior VEGF therapies warrants further study. Clinicians should consider an individualized approach when selecting anti-VEGF agents for patients with a history of IOI, possibly avoiding re-exposure to second-generation agents unless necessary.

## Conclusions

Although second-generation VEGF inhibitors have a greater effect on the resolution of exudation in various retinal diseases, IOI is a major concern after administering second-generation VEGF inhibitors, whereas IOI was rarely seen when a first-generation VEGF inhibitor was administered. Careful monitoring is needed for eyes with a history of IOI when switching from another second-generation VEGF inhibitor to aflibercept (8 mg).

## References

[1] Aldokhail LS, Alhadlaq AM, Alaradi LM, Alaradi LM, AlShaikh FY (2024). Outcomes of anti-VEGF therapy in eyes with diabetic macular edema, vein occlusion-related macular edema, and neovascular age-related macular degeneration: a systematic review. Clin Ophthalmol.

[2] Rosenfeld PJ, Brown DM, Heier JS, Boyer DS, Kaiser PK, Chung CY, Kim RY (2006). Ranibizumab for neovascular age-related macular degeneration. N Engl J Med.

[3] Heier JS, Brown DM, Chong V (2012). Intravitreal aflibercept (VEGF trap-eye) in wet age-related macular degeneration. Ophthalmology.

[4] Dugel PU, Koh A, Ogura Y (2020). HAWK and HARRIER: phase 3, multicenter, randomized, double-masked trials of brolucizumab for neovascular age-related macular degeneration. Ophthalmology.

[5] Heier JS, Khanani AM, Quezada Ruiz C (2022). Efficacy, durability, and safety of intravitreal faricimab up to every 16 weeks for neovascular age-related macular degeneration (TENAYA and LUCERNE): two randomised, double-masked, phase 3, non-inferiority trials. Lancet.

[6] Lanzetta P, Korobelnik JF, Heier JS (2024). Intravitreal aflibercept 8 mg in neovascular age-related macular degeneration (PULSAR): 48-week results from a randomised, double-masked, non-inferiority, phase 3 trial. Lancet.

[7] Hikichi T (2022). Sub-Tenon's capsule triamcinolone acetonide injection to prevent brolucizumab-associated intraocular inflammation. Graefes Arch Clin Exp Ophthalmol.

[8] Shigemoto Y, Sakurada Y, Fukuda Y, Matsubara M, Parikh R, Kashiwagi K (2021). The combination therapy of subtenon triamcinolone acetonide injection and intravitreal brolucizumab for brolucizumab-related intraocular inflammation. Medicine (Baltimore).

[9] Baumal CR, Bodaghi B, Singer M (2021). Expert opinion on management of intraocular inflammation, retinal vasculitis, and vascular occlusion after brolucizumab treatment. Ophthalmol Retina.

[10] Baumal CR, Spaide RF, Vajzovic L (2020). Retinal vasculitis and intraocular inflammation after intravitreal injection of brolucizumab. Ophthalmology.

[11] Inoda S, Takahashi H, Maruyama-Inoue M (2024). Incidence and risk factors of intraocular inflammation after brolucizumab treatment in Japan: a multicenter age-related macular degeneration study. Retina.

[12] Fukuda Y, Sakurada Y, Matsubara M, Kotoda Y, Kasai Y, Sugiyama A, Kashiwagi K (2023). Comparison of one-year outcomes between as-needed brolucizumab and aflibercept for polypoidal choroidal vasculopathy. Jpn J Ophthalmol.

[13] Maruko I, Okada AA, Iida T (2021). Brolucizumab-related intraocular inflammation in Japanese patients with age-related macular degeneration: a short-term multicenter study. Graefes Arch Clin Exp Ophthalmol.

[14] Bourdin A, Cohen SY, Nghiem-Buffet S, Smadja J, Paques M, Fajnkuchen F, Mrejen S (2025). Vitritis following intravitreal faricimab: a retrospective monocentric analysis. Graefes Arch Clin Exp Ophthalmol.

[15] Hosoda S, Sakurada Y, Fukuda Y, Kotoda Y, Kikushima W, Kashiwagi K (2025). Short-term outcomes of three consecutive monthly loading administrations of aflibercept 8 mg for treatment-naïve exudative age-related macular degeneration. Pharmaceuticals (Basel).

[16] Matsumoto H, Hoshino J, Numaga S, Mimura K, Asatori Y, Akiyama H (2024). Retinal vasculitis after intravitreal aflibercept 8 mg for neovascular age-related macular degeneration. Jpn J Ophthalmol.

[17] Hoffmann L, Michels S, Eandi C, Karam MA, Figueiredo EC, Hatz K (2024). Aflibercept high-dose (8mg) related intraocular inflammation (IOI) - a case series. BMC Ophthalmol.

[18] Thangamathesvaran L, Kong J, Bressler SB (2024). Severe intraocular inflammation following intravitreal faricimab. JAMA Ophthalmol.

